# Potential predictability of skipjack tuna (*Katsuwonus pelamis*) catches in the Western Central Pacific

**DOI:** 10.1038/s41598-020-59947-8

**Published:** 2020-02-21

**Authors:** Jihwan Kim, Hanna Na, Young-Gyu Park, Young Ho Kim

**Affiliations:** 1 School of Earth and Environmental Sciences, Seoul National University, Seoul, Republic of Korea; 2 Research Institute of Oceanography, Seoul National University, Seoul, Republic of Korea; 3 Korea Institute of Ocean Science and Technology, Busan, Republic of Korea; 4 Department of Oceanography, Pukyong National University, Busan, Republic of Korea

**Keywords:** Climate sciences, Environmental sciences, Ocean sciences

## Abstract

The Pacific Island countries have a substantial socio-economic dependency on fisheries. Skipjack tuna is one of the most important species in the Western Central Pacific (WCP) and its catches in this region exhibit a spatio-temporal variability influenced by ocean conditions, mainly the El Niño-Southern Oscillation (ENSO). This study investigates the relationship between skipjack tuna catch amounts and environmental variables in the equatorial Pacific during 1990–2014, and evaluates the potential predictability of the catches based on their statistical relationship. A series of regressed and reconstructed spatial patterns of upper-ocean temperature, salinity, currents and precipitation represent ENSO-like variability, and their principal component time series are used to estimate the predictability of skipjack tuna catches in the Federated States of Micronesia (FSM). ENSO-like variability depicted from 100 m temperature and 5 m salinity in the equatorial Pacific exhibit a significant predictability for the annual catch amount in the FSM for several years with a training period of > 20 years. This suggests that the subsurface temperature or near surface salinity can be a better predictor of ecosystem variability than widely used sea surface temperature. Applications of this result to other species could have broad implications for the fishery industry in the WCP.

## Introduction

**Figure 1:**
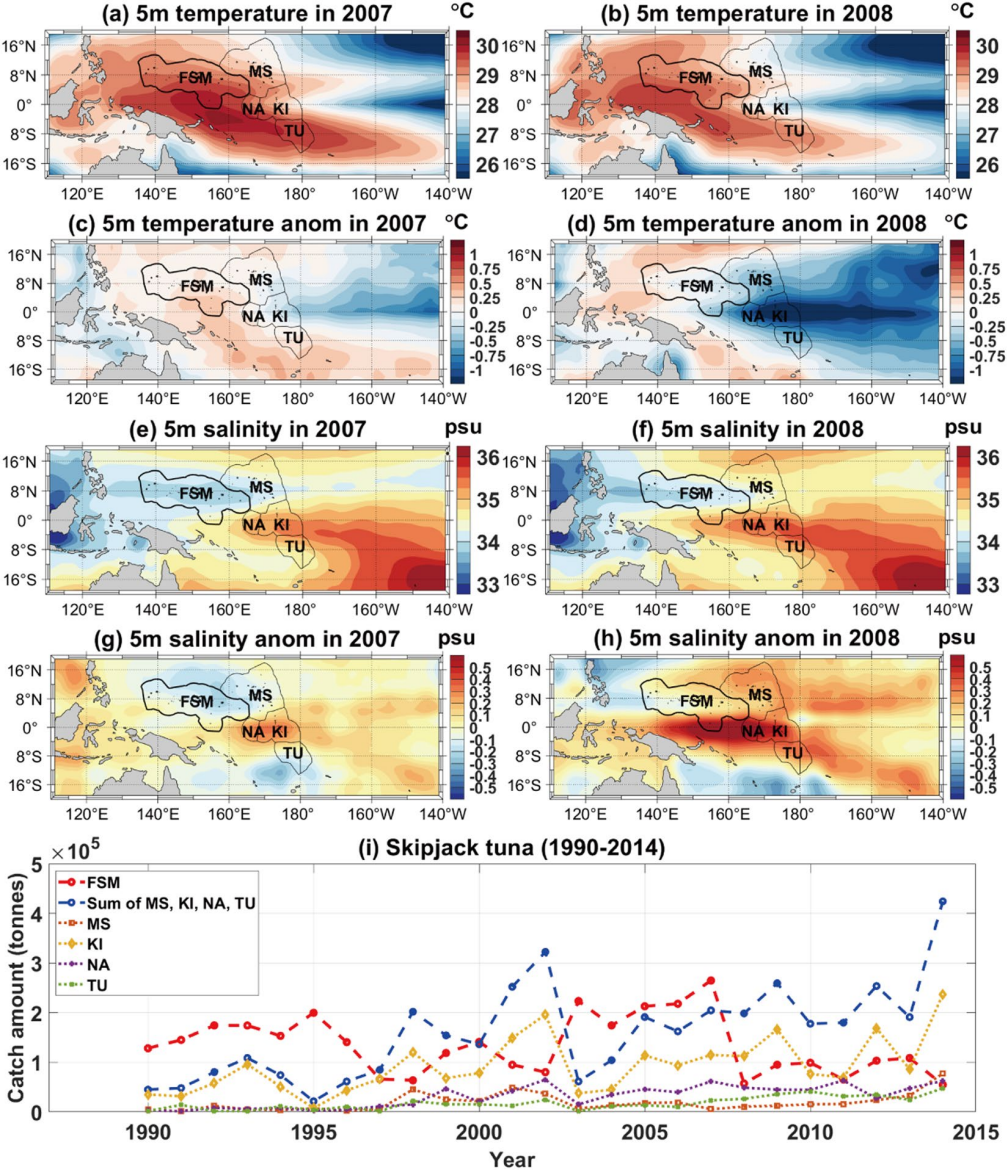
Annual mean temperature and anomalies at 5 m in (**a,c**) 2007 and (**b,d**) 2008 in the Western Central Pacific (WCP). Annual mean salinity and anomalies at 5 m in (**e,g**) 2007 and (**f,h**) 2008 in the WCP. Temperature and salinity anomalies are calculated from the mean of the 1990–2014 period. Black contour lines display the boundaries of the exclusive economic zones (EEZ) of the Federated States of Micronesia (FSM), the Marshall Islands (MS), Kiribati (KI), Nauru (NA), and Tuvalu (TU). (**i**) Annual catch amount of skipjack tuna during 1990–2014 from the FSM (red), MS, KI, NA, and TU (black), in addition to the sum of MS, KI, NA, and TU (blue). Figures in (**a–h**) were created from the analysis of EN4.1.1 with MATLAB R2018a (https://kr.mathworks.com/).

The Pacific Island countries have a crucial dependency on the fishery industry to maintain their economy. Collecting access fees to the exclusive economic zone (EEZ) from foreign fishing fleets contributes considerably to these countries’ financial resources, and the fish processing industry also provides employment to local people^1^. Many of the Pacific Island countries, including the Federated States of Micronesia (FSM), the Marshall Islands, Kiribati (or the Gilbert Islands), Nauru, and Tuvalu, are located in the Western Central Pacific (WCP), a subdivision (statistical area 71) assigned by the Food and Agriculture Organization of the United Nations^2^ (Fig. 1a–h). The annual catch amount of five major species in the WCP from 1950 to 2014 shows that skipjack tuna (*Katsuwonus pelamis*) is generally the most abundant species in recent decades (Supplementary Fig. S1), and that the FSM contributes the most to the annual catches of skipjack tuna among the five EEZs of the WCP (the FSM, the Marshall Islands, Kiribati, Nauru, and Tuvalu) from 1990 to 2014 (Fig. 1i). The combined contribution of the other four EEZs except the FSM is approximately 54% of the total catch from the five EEZs. Figure 1e also shows that the catch amounts of skipjack tuna exhibited different interannual variability in each of the EEZs, displaying sharp increases or decreases in some years. In consideration of the socio-economic importance of the fishery industry in the WCP, it is essential to develop suitable management plans to ensure the sustainability of fisheries and marine resources. In this regard, understanding the present and future availability of the dominant species in each EEZ is a key issue, not only for the Pacific Island countries in the WCP, but also for many other countries that consume fish caught in the EEZs, especially under the conditions of climate change^3–6^.

Skipjack tuna inhabits the upper ocean (shallower than ~100 m) and tends to follow warm water (>28–29 °C)^7,8^. Changes in the habitat of skipjack tuna has therefore been understood to be connected with the zonal displacement of the warm pool in the equatorial Pacific^8^, which is associated with the El Niño-Southern Oscillation (ENSO)^9–12^. The western equatorial Pacific warm pool expands eastward during El Niño periods, whereas it contracts westward during La Niña periods. The spatial distribution of skipjack tuna catches in the WCP is in turn affected by the ENSO because their habitat extends eastwards during the El Niño and contracts westwards during the La Niña^8^. The ENSO variability is also known to be linked with the Inter-Tropical Convergence Zone (ITCZ) and the South Pacific Convergence Zone^13–15^, which are characterized by high rainfall and low sea surface salinity^16–18^. Under the condition of low sea surface salinity, a salinity barrier layer is formed, which prevents thermal convection^19–23^ and contributes to an increase in the near surface temperature that is favorable for the skipjack tuna in the WCP. The ITCZ is reported to shift toward the northeast during the El Niño and toward the southwest during the La Niña periods^24–26^. Investigating the spatial displacement of the western equatorial Pacific warm pool and the ITCZ are beneficial for understanding the interannual variability of skipjack tuna catches in the WCP and its relationship to the ENSO.

The EEZ of the FSM is located in the western part of the WCP. Therefore, the skipjack tuna catch amount in the FSM would be increasing (decreasing) when the warm pool and the ITCZ contract westward (expand eastward) during the La Niña (El Niño)^8^. If this ENSO-dependent availability of skipjack tuna is robust in the FSM on an interannual time scale, then the annual catch amount in the FSM should have a significant negative correlation with ENSO indices (decrease in catch amount during the El Niño). The catch amounts, however, show insignificant correlation coefficients of −0.30 with the Southern Oscillation Index and 0.11 with the Multivariate ENSO Index during the 1990–2014 period. Correlations with Niño 3, Niño 3.4, and Niño 4 indices are also insignificant with correlation coefficients of −0.23, 0.25, and 0.03, respectively. These insignificant correlations can be explained by analysing the relationship between the ENSO-related ocean environment and skipjack tuna catches.

This study investigates this relationship on an interannual time scale by presenting the zonal asymmetry of skipjack tuna catch amounts over the WCP between the FSM, located in the western part of the WCP, and four other countries (Marshall Islands, Kiribati, Nauru, and Tuvalu), located in the eastern part of the WCP. In addition, statistical analysis of the skipjack tuna catches in the FSM and ocean environmental variables (temperature, current velocity, salinity, and precipitation) is used to determine the major physical variables related to the interannual variability of the skipjack tuna catches in the WCP. Finally, the skipjack tuna catch amount in the FSM is presented to be potentially predictable on an interannual time scale based on the statistical relationship between the catch amount and ocean environmental conditions in the equatorial Pacific.

## Results

### Skipjack tuna catches in the FSM: relationship with the ocean environmenta

Figure 1a–h shows the comparison of ocean environmental conditions in the equatorial Pacific between 2007 and 2008, when the catch was the highest and lowest in the FSM respectively during the 1990–2014 period. In 2007, when the catch was the highest, the warm pool and the ITCZ seem to be relatively extended, which is characteristic of the El Niño period, despite ENSO indices indicating a strong La Niña period for the latter half of 2007. Although the extension of the warm pool and the ITCZ is known to decrease the temperature and increase the salinity in the western part of the WCP, the EEZ of the FSM exhibited positive temperature anomalies and negative salinity anomalies in 2007. The highest catch amount and prevalence of more favorable oceanic conditions for skipjack tuna in 2007 supports the hypothesized physical relationship between the catch amount and ocean environmental conditions in the equatorial Pacific, which is not explained by single ENSO indices.

Figure 1i shows the annual catch amounts of skipjack tuna in the EEZs of the FSM and the other four island countries over the WCP from 1990 to 2014. The catches from the FSM and the sum of the other four countries exhibit a negative correlation (*r = *−0.47, *p* = 0.02), which indicates that the catch amount from the FSM increases (decreases) when those of the other four EEZs decrease (increase). When considering the relative geographical position of the FSM and the other four countries (i.e., the FSM to the west and the others to the east), the expansion of the western equatorial Pacific warm pool would cause a shift in the habitat of skipjack tuna eastwards, thereby contributing to an increase in the catch amounts in the eastern part of the WCP and a simultaneous decrease in the catch amount from the FSM. This zonal asymmetry observed in the WCP suggests that it would be helpful to understand the interannual variability of the catch amounts in the FSM for estimating the catch amounts in the eastern part of the WCP.

**Figure 2:**
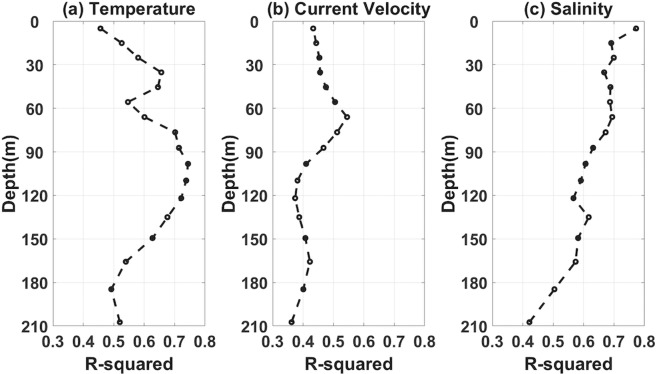
R-squared values of the regression at each depth with respect to the skipjack tuna catch amounts in the Federated States of Micronesia (FSM) from 1990 to 2014 for (**a**) temperature, (**b**) current velocity, and (**c**) salinity.

As mentioned, single ENSO indices do not fully delineate the interannual variability of the catch amounts in the FSM. The ocean environmental conditions associated with the catch variability is derived from a statistical analysis based on the cyclostationary empirical orthogonal function (CSEOF)^27,28^. The regression analysis was conducted using the CSEOF principal component (PC) time series of each variables, i.e. temperature, current velocity, salinity, and surface precipitation at each depths of the upper 200 m, with the exception of surface precipitation, in the equatorial Pacific Ocean (30°S–30°N, 100°E–60°W) (refer to Data and Methods for details). This analysis revealed that the variables exhibit different R-squared values of regression with respect to the skipjack tuna catch amounts at different depths in the FSM (Fig. 2). Generally, temperature and salinity display higher R-squared values in comparison to those of current velocity. Subsurface temperature at ~100 m and near surface salinity present stronger statistical relationships with the target time series, the interannual variability of skipjack tuna catches in the FSM (red line in Fig. 1i). Note that the near surface temperature exhibits lower R-squared values than the subsurface temperature and surface salinity. The different depths of maximum R-squared values for the different variables suggest that the regression results can reflect both/either the favorable habitat of skipjack tuna and/or the representability of the particular variable at a particular depth.

**Figure 3:**
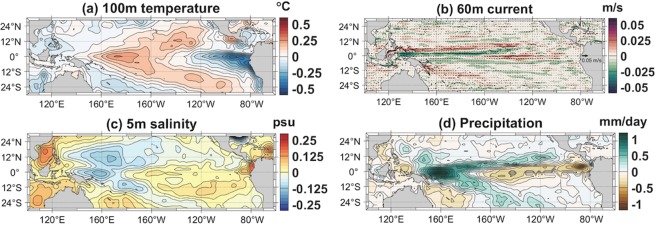
Regressed and reconstructed anomalies of ocean environmental variables in the equatorial Pacific Ocean with respect to the skipjack tuna catch amount in the Federated States of Micronesia (FSM) from 1990–2014: (**a**) temperature at 100 m, (**b**) current velocity at 60 m (shading: zonal velocity), (**c**) salinity at 5 m, and (**d**) precipitation. Figures in (**a,c**), (**b**), and (**d**) were created from the analysis of EN4.1.1, SODA 3.4.2, and CMAP, respectively, with MATLAB R2018a (https://kr.mathworks.com/).

Figure 3 shows the regressed and reconstructed anomalies of the 100 m temperature, 60 m current velocity, 5 m salinity, and surface precipitation. Thus, the spatial patterns in Fig. 3 are related to the interannual variability of the skipjack catch in the FSM with the corresponding R-squared values. The depths of each of the variables (except precipitation) are selected to be displayed based on their high R-squared values (100 m temperature: 0.75; 60 m current velocity: 0.53; 5 m salinity: 0.77; precipitation: 0.55). 100 m temperature anomalies show positive values in the western to central equatorial Pacific and negative values in the eastern equatorial Pacific. The regression results indicate that, under this La Niña-like ocean condition^9,10^, tuna catches increase in the FSM and decrease in the eastern part of the WCP. The 60 m current velocity present westward anomalies along the equator, which also indicates La Niña-like conditions. Negative anomalies of the 5 m salinity in the western equatorial Pacific and positive anomalies in the eastern equatorial Pacific suggest a westward shift of the ITCZ that is linked to the La Niña^26^. The westward shift of the ITCZ can also be observed in the precipitation anomalies, which display consistent spatial patterns similar to those of the 5 m salinity. All the ocean environmental variables at different depths are independently regressed with respect to the annual catch amounts of skipjack tuna in the FSM and exhibit La Niña-like ocean conditions; thus, indicating that the interannual variability of the catches is associated with an ENSO-like variability.

### Predictability of catch amounts

**Figure 4:**
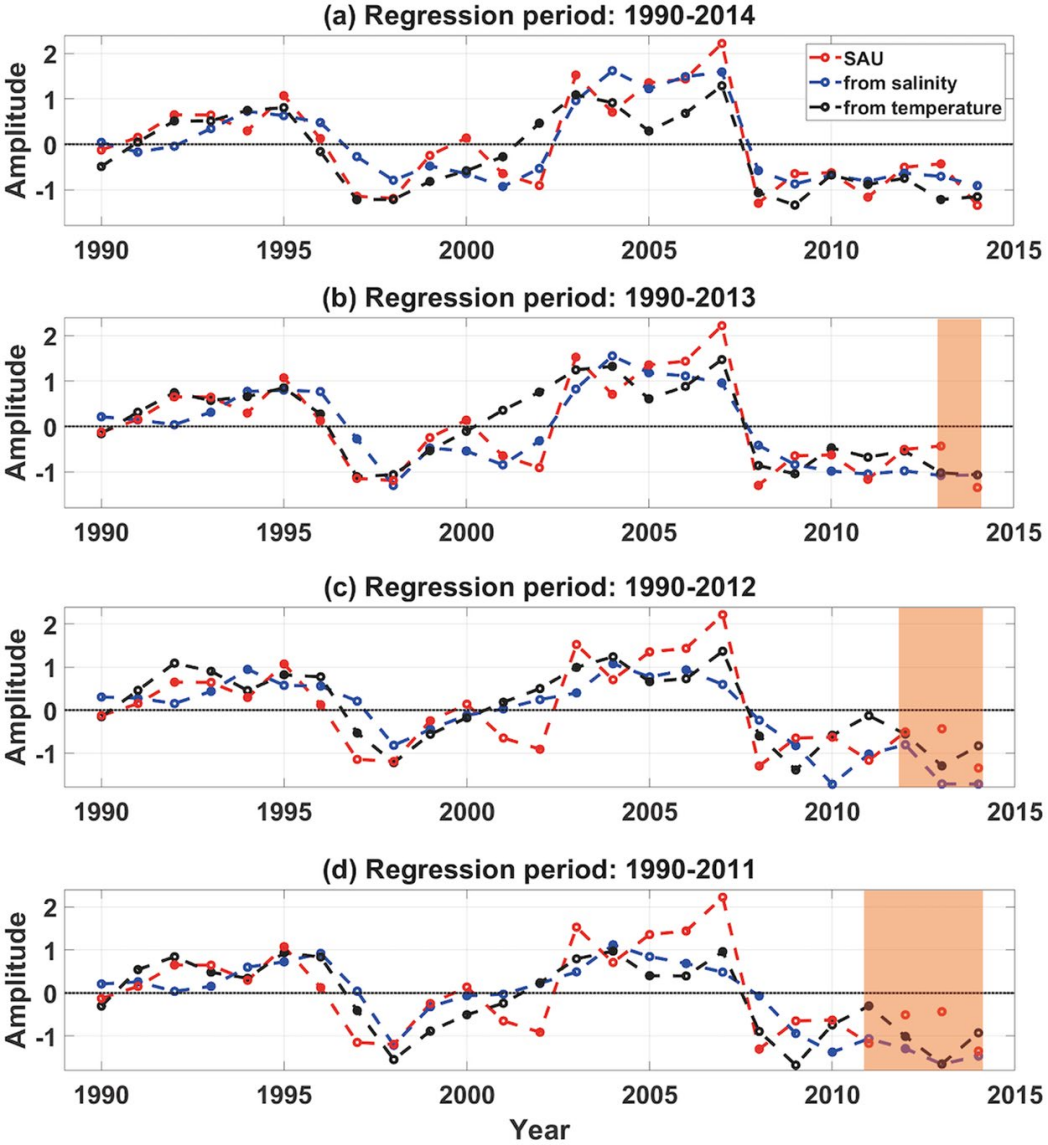
Observed (red), estimated, and predicted annual catch amount of skipjack tuna in the Federated States of Micronesia (FSM) based on the relationship with the 5 m salinity (blue) and 100 m temperature (black): (**a**) regression during 1990–2014, (**b**) regression during 1990–2013 and prediction for 2014, (**c**) regression during 1990–2012 and prediction for 2013–2014, and (**d**) regression during 1990–2012 and prediction for the 2012–2014 period. Prediction period is shaded in orange.

The statistical relationship between the skipjack tuna catches in the FSM and the ENSO-like variability (Figs. 2 and 3) can be used to predict the catch amount if information on the ocean environment is given, particularly that of the 100 m temperature or 5 m salinity in the equatorial Pacific Ocean. Figure 4a shows the regression results for the entire 25-year period of the dataset (1990 to 2014). Correlation coefficients between the observed annual catch amounts and those estimated from the 100 m temperature and 5 m salinity are 0.87 and 0.88, respectively. By conducting a regression analysis of the temperature or salinity targeting on the catch amount for a limited time period (i.e., shorter than the total length of the dataset), which is termed the training period, the catch amount can be predicted for the remaining years and the performance can be evaluated for the prediction years. Figure 4b–d presents the regression relationship obtained from different training periods and their application for annual predictions up to 3 years. The 1-year prediction based on the regression relationship for a 24 year training period (Fig. 4b) exhibits plausible results; the correlation coefficients for the entire period (24-year estimation plus 1-year prediction) based on the 100 m temperature and 5 m salinity are 0.84 and 0.83, respectively. The predictability based on 23- and 22- year training periods also show acceptable results, although the actual values are higher than the predicted catch amounts (Fig. 4c,d).

## Discussion

One of the most successful fishing grounds for skipjack tuna is located in the convergence zone between the warm pool and equatorial upwelling in the central Pacific where the warm, low-salinity water and the cold, saline water converge in the upper ocean^7,8^. It should be pointed out that, however, that this analysis is not based on a prior understanding of the physiological conditions of skipjack tuna caught in the equatorial Pacific. Rather, the higher R-squared values of the specific variables at specific depths (Fig. 2) suggest that they would be associated with favorable conditions for skipjack tuna, for example, regions with an enhanced availability of food, sufficient plankton, and an accumulation of preferred prey. The warm pool, however, is known to be oligotrophic with low chlorophyll and primary production, which can be counter-intuitive^8^. Studies have indicated that chlorophyll may be advected to the warm pool region by the westward current during the transition phase of the ENSO in the equatorial Pacific^29–31^. Regression analysis of surface chlorophyll, conducted only from 1998 to 2014 only due to the shorter length of the available chlorophyll dataset, indeed exhibits a relatively high R-squared values of 0.76 (not shown). This suggests that sea surface chlorophyll variability could also be a potential predictor when longer data is available to obtain a robust statistical relationship.

Most of the climate indices associated with ENSO are computed using sea surface temperature. It is notable that the low correlation, thus low predictability, between the single ENSO indices and the catch amount is explained by the lower R-squared values of the sea surface temperature compared to those of the subsurface temperature. The higher R-squared values of the subsurface temperature compared to that of surface temperature would be partly due to the larger temporal variability of the subsurface temperature (Supplementary Fig. S2). The smaller amplitude of temperature anomalies at the sea surface may not be great enough to induce physiological or behavioral responses of the skipjack tuna. Indeed, the surface salinity exhibits a larger temporal variability and a higher R-squared value with the catch amount compared to the subsurface salinity (Supplementary Fig. S2). Furthermore, the present study highlights that surface salinity can provide a better basis for predicting the skipjack tuna catch amount in the FSM than surface temperature, possibly because the amplitude of sea surface temperature anomalies tends to decrease rapidly due to active heat exchanges between the ocean and the atmosphere.

It should be pointed out that although the subsurface temperature and surface salinity are better at prediction, relying on these variables would not always be the best option for an operational application due to their limited availability and/or accuracy over longer time periods. An alternative option would be to obtain a statistical relationship between the subsurface variability and the surface temperature from a long-term best available dataset, and then apply the relationship to predict the target variability from the surface temperature.

The predictability presented in this study is based on the statistical relationship between the annual catch amounts and the ocean environmental variability in the equatorial Pacific Ocean. Future prediction is applicable if future ocean environmental data is available which is the case particularly for the equatorial Pacific Ocean, though the quality and perspective vary. Additionally, the prediction can be conducted if the PC time series of ocean environmental variability is temporally extended by using an autoregressive model^32^ or other similar methods. The prediction error mainly depends on how well the selected variables represent the ocean environmental variability that is associated with the target variability, assuming that the abundance of skipjack tuna is well represented by the target time series. If anthropogenic effects such as illegal fishing severely impact the skipjack population, the regression error will increase because the target time series itself cannot fully reflect the natural variability of the catch amount. However, the statistical significance of the regression relationship determined in this study indicates that the target time series can represent the skipjack tuna variability during the training period.

Although a statistical relationship generally becomes more robust when the analysis is conducted using a longer dataset, the inclusion of catch data prior to the 1990s is not helpful for increasing the predictability. This is because temporal changes in the anthropogenic effect, which are not depicted in the ocean environment, would be larger. However, if the anthropogenic effect is something that is depicted in the ocean environment (e.g. if the temperature and salinity variability presented in Fig. 3 somehow occurs more frequently in the future due to climate change induced by anthropogenic effect), then the prediction would tell catch amount of skipjack tuna would increase in the FSM. Future projections of ocean environmental variability using numerical models (e.g. the Couple Model Intercomparison Project) can provide useful information for the sustainable fishery industry in the WCP, particularly under the conditions of climate change.

## Data and Methods

### Fishery data

Annual catch amounts of fish in each of the EEZs in the WCP were obtained from Sea Around Us (SAU, http://www.seaaroundus.org), which combines officially reported data to the Food and Agriculture Organization of the United Nations (http://www.fao.org/fishery/statistics/en) as well as unreported data, including major discards^33,34^. The EEZ-based annual dataset was compared with a spatially gridded monthly dataset provided by the Western Central Pacific Fisheries Commission (WCPFC, https://www.wcpfc.int/public-domain). The WCPFC data includes the catch per unit of effort (CPUE) information from commercial fisheries to measure the relative abundance of fish stocks and uses location information from tagged global positioning system devices. The comparison between SAU’s catch amount for the EEZ and the WCPFC’s CPUE of the corresponding grids revealed a similar temporal variability, although SAU’s data showed larger values (Supplementary Fig. S3). In this study, the predictability of the skipjack tuna catch amount in the FSM was examined using the SAU EEZ dataset due to the relatively low spatial resolution (5°) and inconsistent number of empty grids in time in the gridded dataset. The autocorrelations of the target time series (SAU annual mean catch amount in the FSM) exhibit a sharp decrease with time, and the e-folding time scale is approximately one year (not shown).

### Ocean environmental data

Monthly mean values of upper-ocean (5–200 m) temperature and salinity were acquired from EN4.1.1, and those of current velocity were obtained from Simple Ocean Data Assimilation (SODA) 3.4.2. Surface precipitation data were obtained from CPC Merged Analysis of Precipitation (CMAP). The EN4.1.1 dataset is spatially gridded based on quality-controlled *in situ* vertical profiles of temperature and salinity^35^. The SODA 3.4.2 dataset is a reanalysis product with ERA-Interim forcing and the National Oceanic and Atmospheric Administration (NOAA)/Geophysical Fluid Dynamics Laboratory CM2.5 coupled model^36^. The CMAP dataset is a merged product of precipitation estimates from satellites, gauges, and NCEP/NCAR reanalysis^37^. All ocean environmental datasets were analyzed from 1990 to 2014 over the equatorial Pacific Ocean (30°S–30°N, 100°E–60°W) to determine the statistical correlations between the ocean environmental variability and the skipjack tuna catch amounts. The Copernicus-GlobColor surface chlorophyll data was obtained from the Copernicus Marine Environmental Monitoring Service for the 1998–2014 period. The daily surface chlorophyll data was reproduced based on satellite observation from SeaWiFs, MODIS-Aqua, MERIS, VIIRS-N, and OLCI-S3A^38^.

### CSEOF and regression analysis

The ocean environmental data, including temperature, salinity, current velocity, and precipitation, were analyzed by using the CSEOF technique^27,28^. Each variable at each depth, *D*(*r*, *t*), was decomposed into cyclostationary loading vectors (CSLVs), *E*_*n*_(*r*, *t*), which illustrate the spatio-temporal evolution of the *n*th mode, and their corresponding PC time series, *P*_*n*_(*t*), as defined by Eq. (1):

1 $$D(r,t)={\sum }_{n}{E}_{n}(r,t){P}_{n}(t),$$

where *n*, *r*, and *t* denote the mode number, space, and time, respectively. Note that current velocity was analyzed as one variable including both zonal and meridional components by shuffling the two components. CSLVs are periodic with the nested period, *d*, which was set to 12 months, such that each *n*th mode, *E*_*n*_(*r*, *t*), consisted of 12 monthly spatial patterns as expressed by Eq. (2):

2 $${E}_{n}(r,t)={E}_{n}(r,t+d).$$

The statistical relationship between the ocean environmental variability and the annual catch amount of the skipjack tuna was obtained by conducting regression analysis in the CSEOF space^39^, as described by Eq. (3):

3 $$T(t)\approx {\sum }_{m=1}^{10}{\alpha }_{m}{\tilde{P}}_{m}(t),$$

where ${\tilde{P}}_{m}(t)$ is the annual mean of the *m*th mode of the PC time series for each of the ocean environmental variables and *T*(*t*) is the target time series of the observed skipjack tuna catch amount in the FSM. The first ten CSEOF PC time series of the predictors were used for the regression analysis as more modes would increase the chance of overfitting, which should be avoided for a stable predictability. Note that the regression was conducted using the annual mean PC time series, not the original monthly PC time series, because the target time series, *T*(*t*), was available as annual means. By using the regression coefficients, *α*_*m*_, regressed and reconstructed spatio-temporal patterns, *R*(*r*, *t*) were generated, as expressed by Eq. (4):

4 $$R(r,t)={\sum }_{m=1}^{10}{\alpha }_{m}{E}_{m}(r,t).$$

Thus, the resulting ocean environmental evolution, *R*(*r*, *t*), shares the long-term variability of the fishery data, *T*(*t*), with some regression error. Annual means of *R*(*r*, *t*) are presented for one representative depth for each variables in Fig. 3, and the original 12 sets of *R*(*r*, *t*) are displayed for the 100 m temperature and 5 m salinity in Supplementary Figs. S4 and S5.

The prediction was conducted using the statistical relationship *α*_*m*_during the training period, *L*, within the total length of the time series of the regression analysis. The estimated and predicted annual catch amount $\hat{T}(t)$, including the prediction period, *R* can be written as Eq. (5)^40^:

5 $$\hat{T}(t)={\sum }_{m=1}^{M}{\alpha }_{m}{\tilde{P}}_{m}(t),\,\,\,\,{\rm{t}}\in {\rm{L}}+{\rm{R}}.$$

In Fig. 4, the training period, *L*, is 25, 24, 23, or 22 years and the prediction period, *R*, is 0, 1, 2, or 3 years, respectively. The number of modes used in the regression was 10. Note that the regression error during the training period in Eq. (3) would decrease when the number of modes for the regression increases (i.e., a larger *m*). However, this does not guarantee a better predictability during the prediction period, *R*.

## Supplementary information

Supplementary information.

## Data Availability

All data generated or analysed during this study are included in this published article (and its Supplementary Information files) or available from the corresponding author on reasonable request.
