# Supplementary material for: Potential predictability of skipjack tuna (Katsuwonus pelamis) catches in the Western Central Pacific
Source: Sci Rep. 2020 Feb 21;10:3193. doi: 10.1038/s41598-020-59947-8 (PMC7035267; doi:10.1038/s41598-020-59947-8)
Supplement: Supplementary file 1 — Supplementary information. [file 41598_2020_59947_MOESM1_ESM.pdf]

Supplementary information

Potential predictability of skipjack tuna (*Katsuwonus pelamis*) catches in the Western Central Pacific

Jihwan Kim[1], Hanna Na[1,2*], Young-Gyu Park[3], and Young Ho Kim[4]

[1]School of Earth and Environmental Sciences, Seoul National University, Seoul, Republic of Korea

[2]Research Institute of Oceanography, Seoul National University, Seoul, Republic of Korea

[3]Korea Institute of Ocean Science and Technology, Busan, Republic of Korea

[4]Department of Oceanography, Pukyong National University, Busan, Republic of Korea

[*]Corresponding author: Hanna Na (hanna.ocean@snu.ac.kr)

School of Earth and Environmental Sciences/Research Institute of Oceanography, Seoul National University, Seoul 08826, Republic of Korea

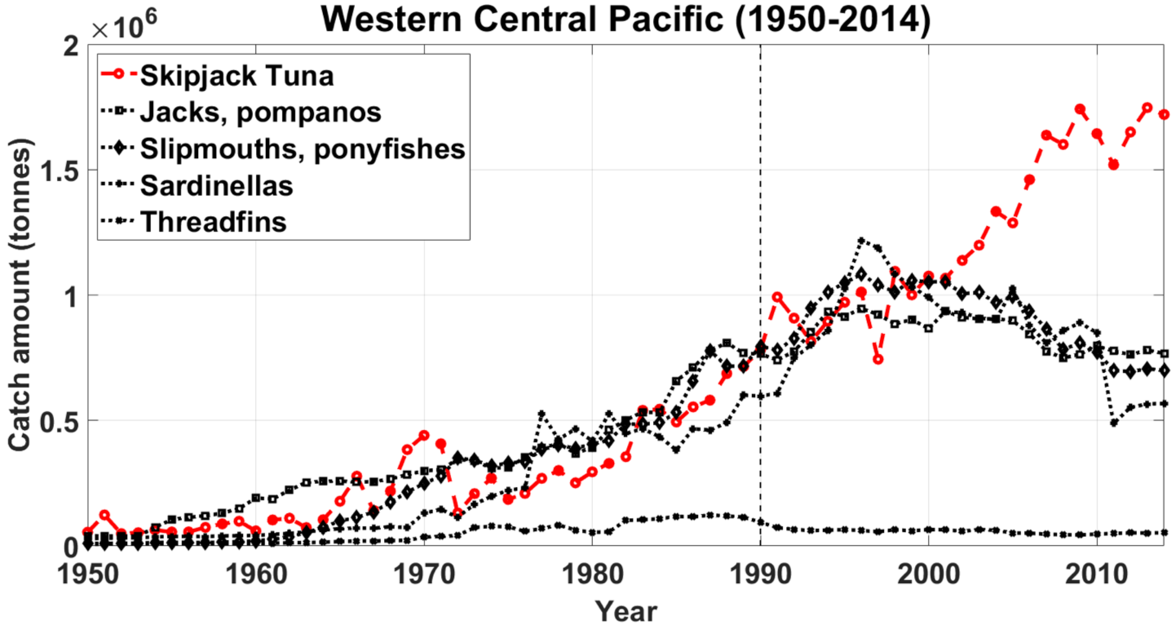

Supplementary Figure S1. Annual catch amount of the five-dominant species of fish (skipjack tuna, jacks, slipmouths, sardinellas, and threadfins) in the Western Central Pacific from 1950 to 2014.

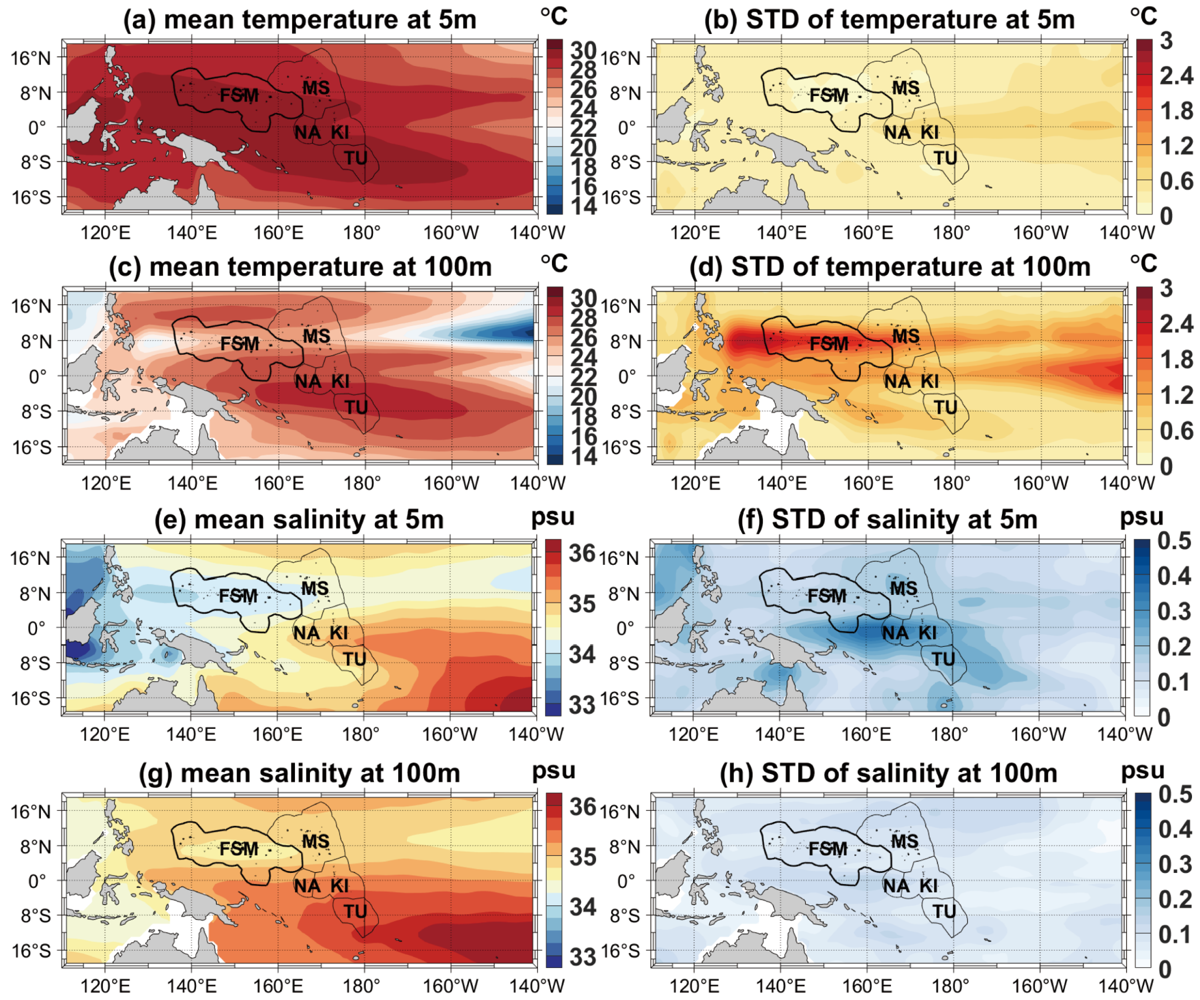

Supplementary Figure S2. Mean (left panel) and standard deviation (right panel) of the annual mean temperature and salinity at 5 m and 100 m during 1990–2014: (**a,b**) 5 m temperature, (**c,d**) 100 m temperature, (**e,f**) 5 m salinity, and (**g,h**) 100 m salinity. Figures were created from the analysis of EN4.1.1 with MATLAB R2018a (https://kr.mathworks.com/).

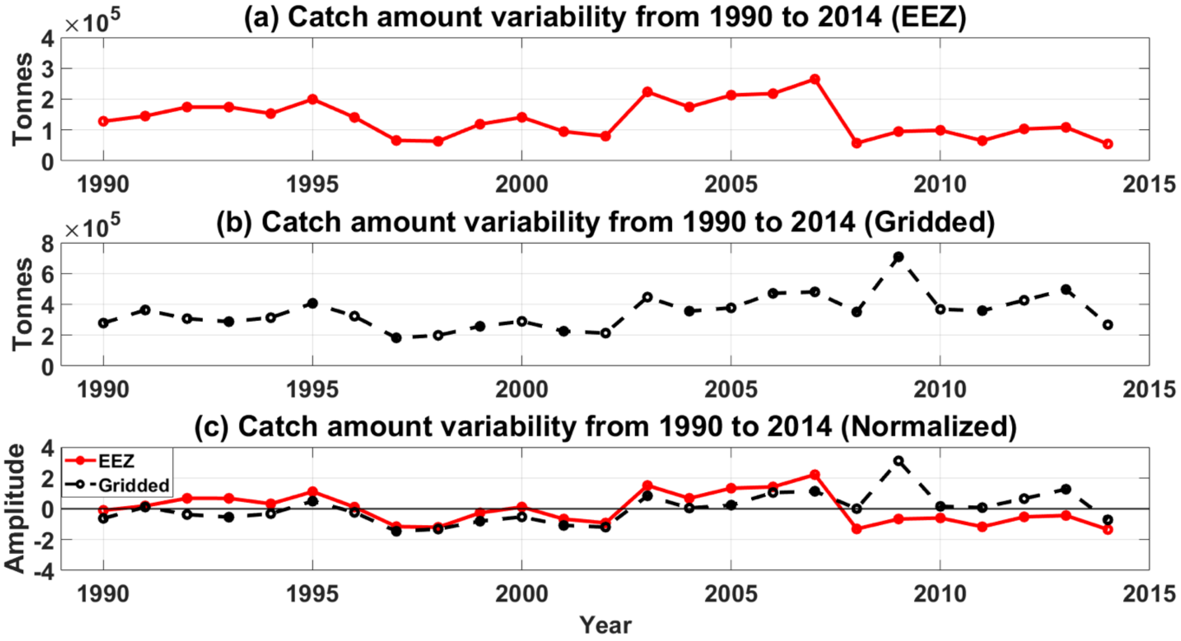

Supplementary Figure S3. Comparison of annual catches of skipjack tuna in the Federated States of Micronesia (FSM) during 1990–2014 from (**a**) Sea Around Us (SAU) exclusive economic zones (EEZ) data (red solid line), (**b**) Western Central Pacific Fisheries Commission (WCPFC) gridded data (black dotted line), and (**c**) normalized data.

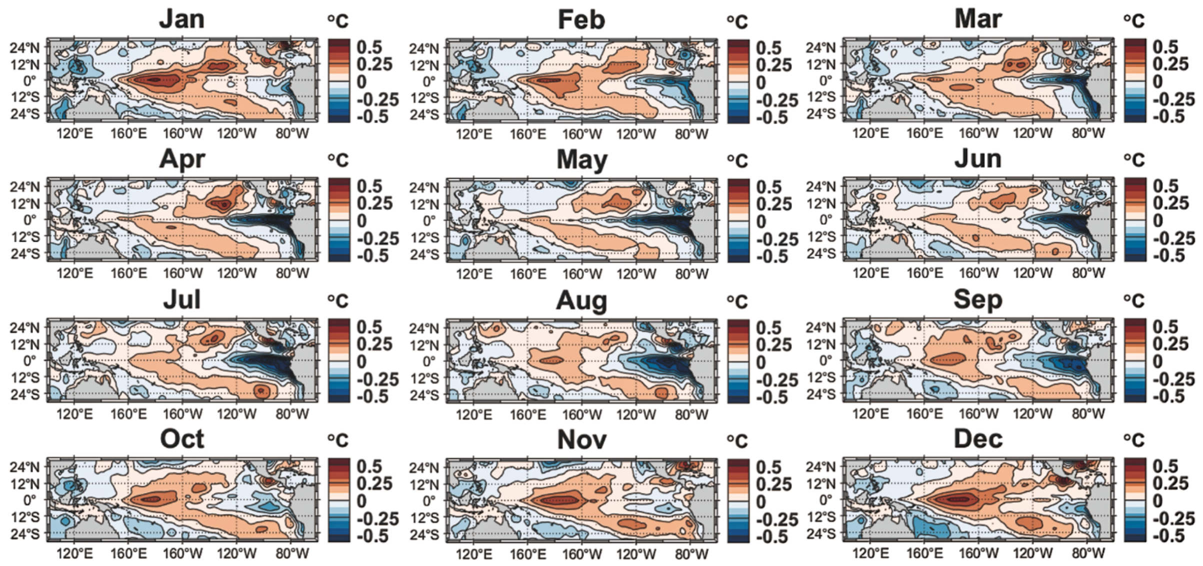

Supplementary Figure S4. Monthly regressed and reconstructed anomalies of ocean temperatures at 100 m in the equatorial Pacific Ocean with respect to the skipjack tuna catch amount in the Federated States of Micronesia (FSM) during 1990–2014. The mean of the monthly anomalies is shown in Figure 3a. Figures were created from the analysis of EN4.1.1 with MATLAB R2018a (https://kr.mathworks.com/).

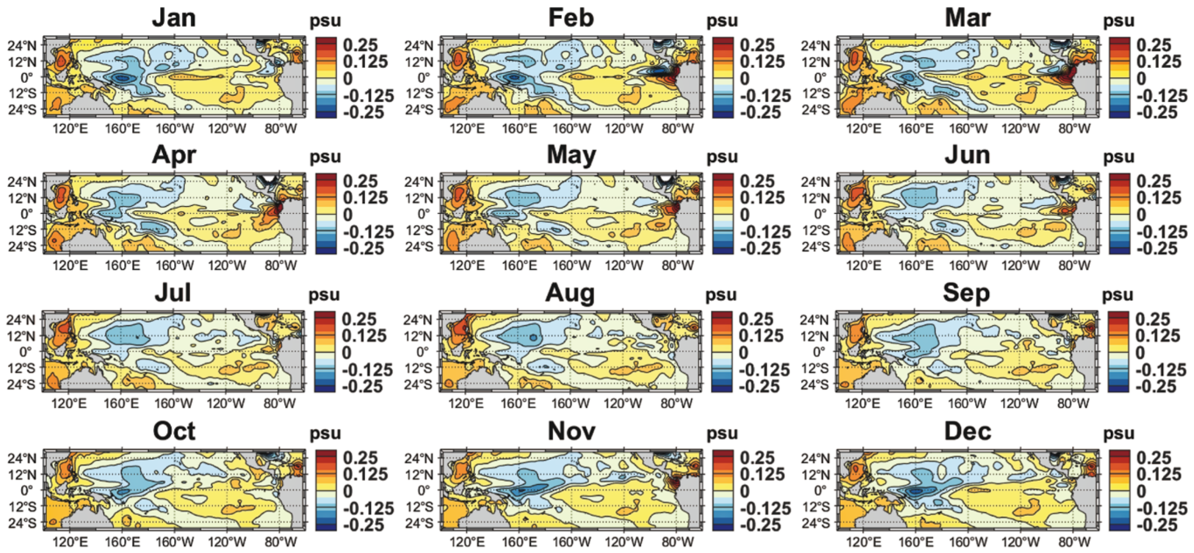

Supplementary Figure S5. Monthly regressed and reconstructed anomalies of salinity at 5 m in the equatorial Pacific Ocean with respect to the skipjack tuna catch amount in the Federated States of Micronesia (FSM) during 1990–2014. The mean of the monthly anomalies is shown in Figure 3c. Figures were created from the analysis of EN4.1.1 with MATLAB R2018a (https://kr.mathworks.com/).
